# A Hidden Surgical Challenge: Ureterovaginal Fistula Encountered During Vesicovaginal Fistula Repair

**DOI:** 10.7759/cureus.90664

**Published:** 2025-08-21

**Authors:** Sushmitha Kothapalli, Venkateshen Palanisamy, Velmurugan Palaniyandi, Hariharasudhan Sekar, Sriram Krishnamoorthy

**Affiliations:** 1 Urology, Sri Ramachandra Institute of Higher Education and Research, Chennai, IND

**Keywords:** contrast-enhanced computed tomography (cect), cystoscopy, double-j stent, fistula, hysterectomy, retrograde pyelogram, ureteric reimplantation, urine leak, uvf, vvf

## Abstract

The concurrent occurrence of vesicovaginal and ureterovaginal fistulas following hysterectomy is rare, accounting for less than 1% of post-hysterectomy urinary fistulas, leading to significant physical and psychological distress.

We present the case of a 36-year-old woman referred for evaluation of continuous urinary leakage following laparoscopic hysterectomy for fibroid uterus, with an initial clinical suspicion of vesicovaginal fistula (VVF). However, a comprehensive diagnostic assessment using multiple imaging and endoscopic modalities revealed an occult right ureterovaginal fistula (UVF). This finding highlights the growing need for a thorough preoperative evaluation to avoid missed diagnoses and ensure appropriate surgical planning. She underwent simultaneous right ureteric reimplantation and vesicovaginal fistula repair, resulting in complete resolution of urinary leakage. Her postoperative course was uneventful, with satisfactory recovery and no recurrence of symptoms during follow-up.

This case underscores the critical importance of meticulous preoperative evaluation in patients presenting with VVF. A high index of clinical suspicion, combined with appropriate imaging modalities, is essential to identify any coexisting UVF and ensure accurate, comprehensive surgical planning. Failure to detect and address a concomitant UVF during the initial VVF repair may result in persistent urinary leakage, despite an otherwise successful procedure. Long-term postoperative follow-up is crucial to monitor for delayed complications and ensure sustained clinical resolution.

## Introduction

A vesicovaginal fistula (VVF) is an abnormal epithelialized communication between the urinary bladder and the vaginal canal. This pathological connection results in continuous involuntary urine leakage into the vagina, which may occur independently or alongside voluntary micturition [1]. The condition significantly affects the patient’s quality of life and often requires surgical intervention for definitive management.

VVF causes significant distress, morbidity, and psychosocial problems for all women affected by this condition [2]. VVF often shows up within a week to ten days after pelvic surgery or after gynaecological, obstetric, or urological procedures; hysterectomy is the most common cause [3]. Additional potential causes include radiotherapy, chronic infections, inflammatory conditions, neglected pessaries, and sexual trauma [3]. Successful surgical management of VVF is essential in alleviating distressing symptoms, the restoration of continence, and improvement in quality of life. An often-overlooked challenge in VVF management is the potential presence of a concurrent ureterovaginal fistula (UVF), which arises in about 10% of cases [4].

Ureterovaginal fistulas (UVF) are abnormal connections between the ureter and vagina. They often happen after gynaecological procedures, such as total abdominal hysterectomies [5]. UVF's aetiology can be multifactorial. Gynaecological surgery, especially TAH, is still the most frequent iatrogenic reason, for instance, anterior wall prolapse and urethral diverticulum repair [1,3]. It could frequently present post-operation through continuous urinary leakage, the consistent urge to void, as well as a certain amount of flank pain along with discomfort [4,6].

VVF are categorised by size as simple, intermediate, or complex fistulae. Fistulas measuring less than 0.5 cm that do not indicate malignancy are simple [7], but fistulas greater than 2.5 cm that show chronic disease [2], a history of failed repairs, or other associated fistulas like UVF or rectovaginal fistulas are complex. Fistulas measuring between 0.5 and 2.5 cm in size are frequently complex [8].

Ureterovaginal fistulas (UVFs) can be classified by etiology, anatomical location, timing of presentation, and complexity. Most are iatrogenic, typically following gynecologic surgery, though obstetric trauma, pelvic malignancy, radiation injury, infection, and pelvic trauma are also recognized causes [5, 7]. Anatomically, UVFs are described as involving the upper, middle, or lower third of the ureter, with distal injuries being the most common after hysterectomy. By timing, they may be early, presenting within six weeks of injury, or delayed, appearing later due to ischemia or fibrosis. In terms of complexity, they may be simple - single tract with healthy margins and no associated vesicovaginal fistula - or complex, with multiple tracts, concomitant fistulas, radiation damage, malignancy, or dense fibrosis [9].

A preliminary assessment of fistulae is important to identify the most suitable repair procedure [3]. Diagnosing small or complex fistulas can be arduous. Advanced imaging techniques, like contrast-enhanced computed tomography, are valuable in detecting VVFs and associated injuries [9]. Intraoperative retrograde pyelography is extremely useful in identifying ureteral injuries that may not be apparent on preoperative imaging.

The literature provides evidence on various treatment options for UVF, including conservative approaches involving catheterisation or electrocoagulation [2] and minimally invasive surgical treatments like the placement of double J stents [6]. When a contemporaneous UVF is not discovered before or during VVF repair, the patient will continue to experience urine leakage despite successful VVF closure, which can lead to misinterpretation of the surgical outcome and unnecessary misery for both the patient and the treating physician.

Not identifying an associated UVF in a patient undergoing VVF repair has significant consequences. It is detrimental to the surgical outcome if there is persistent urinary leakage postoperatively. This, however, calls for other investigations, more morbidity, and, finally, a second surgical intervention. This secondary procedure is usually more complicated due to previous surgical scarring, altered anatomy, and the possibility of progressive ureteral obstruction or renal dysfunction in the absence of treatment for an extended period [5]. Also, undiagnosed UVF may lead to hydroureteronephrosis, urinary tract infection, and reduced renal function, making managing the patient difficult. Since the consequences of missing a UVF are rather severe, this critical dyad should be evaluated systemically and aggressively as part of the preoperative assessment of any patient with VVF. The detection of a coexisting UVF allows for its management during the same surgery, thus preventing unnecessary morbidity and maximizing surgical results [7]. Additional diagnostic methods, such as MRI urography, can provide imaging in patients with impaired renal function when contrast imaging may be contraindicated. Cystoscopy is useful for evaluating bladder involvement and identifying small fistulas that may be missed on imaging.

## Case presentation

We report the case of a 36-year-old woman who presented with persistent urinary leakage per vagina for 10 days, following a total abdominal hysterectomy with bilateral salpingo-oophorectomy performed under general anaesthesia for a fibroid uterus, with histopathology confirming benign pathology. The Foley catheter was removed on postoperative day (POD) 3; she voided normally, but by POD 10, she developed continuous urinary dribbling per vagina. She was referred to us with a diagnosis of VVF on POD 15. Upon review, she gives a history of the urge to void and passing approximately 100-150 ml during normal voiding.

Clinical examination revealed continuous urinary leakage from the vaginal canal, with normal external genitalia, skin excoriation, and a normal vaginal opening without bleeding or cystocele. Palpation identified a firm area on the anterior vaginal wall with mild mucosal scarring. Despite being catheterised, she had a persistent leak.

**Figure 1:**
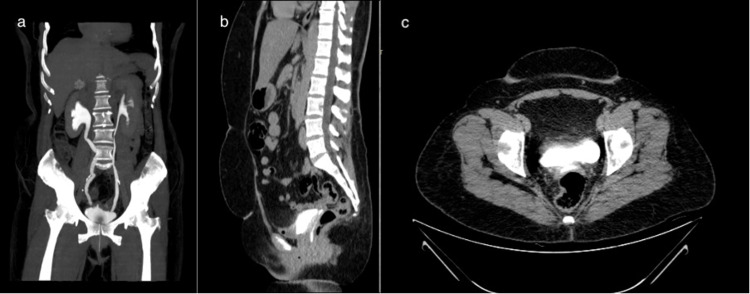
Multiplanar CT evaluation of the abdomen and pelvis: coronal, sagittal, and axial views. (a) Coronal reconstruction showing right moderate hydronephrosis. (b) Sagittal section (delayed phase) of CECT demonstrating contrast leak from bladder into vaginal canal. (c) Cross section (delayed phase) of CECT demonstrating contrast leak from bladder into vaginal canal.

**Figure 2:**
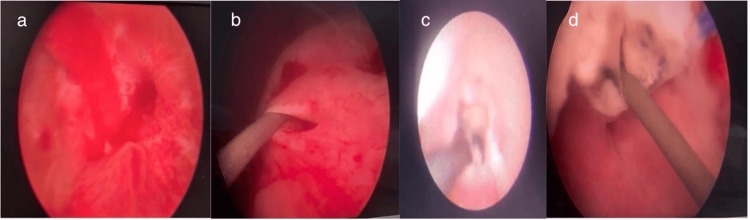
Endoscopic evaluation of urogenital fistula with ureteroscopy and vaginoscopy findings. (a) Cystoscopy image - Mucosal defect in the left posterior bladder wall. (b) Cystoscopy image - Right ureteric orifice with guidewire cannulation. (c) Right ureterorenoscopy image - guidewire with mucosal tear at the distal ureter and (d) Vaginoscopy image - showing guidewire within the fistulous tract

A contrast-enhanced computerized tomography (Figure 1) revealed a small defect in the posterior bladder wall with contrast leakage into the vagina. Focal wall thickening was observed at the right vesicoureteral junction, suggesting likely inflammatory changes. Furthermore, the bulky right kidney with oedematous cortex and moderate hydroureteronephrosis indicated a likelihood of secondary obstructive features due to the vesicoureteral junction wall thickening (Figure 2). No free peritoneal contrast leakage was noted. These findings raised the suspicion of an associated UVF.

The patient underwent cystoscopy under local anaesthesia at our cystoscopy suite. The findings were a 1 cm mucosal breach in the left posterior bladder wall, supratrigonal with surrounding unhealthy tissue (Figure 2a) confirming the diagnosis of VVF. However, we also noticed normal efflux from the left ureteric orifice but absent efflux from the right ureteric orifice. This further strengthened our suspicion of a missed UVF; hence, the patient was planned for cystoscopy under anaesthesia.

**Figure 3:**
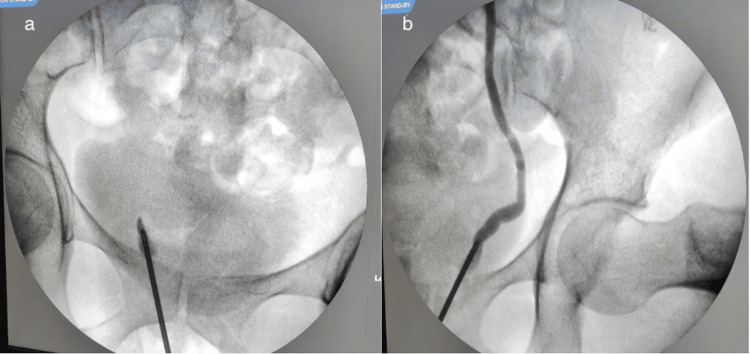
Intraoperative retrograde pyelography (RGP) performed to assess ureteric integrity. (a) Non-visualized right ureter with contrast spilling over into the urinary bladder. (b) Intact left ureter.

Attempts to cannulate the right ureter with a guidewire were unsuccessful (Figure 2b), right ureterorenoscopy showed mucosal disruption at the distal end (Figure 2c), and the guidewire was observed in the vaginal canal (Figure 2d), confirming UVF. Retrograde pyelography showed no contrast in the right distal ureter (Figure 3a), while the left ureter had a normal appearance (Figure 3b).

Under anaesthesia, cystoscopy confirmed VVF and absent right ureteric efflux. In the same sitting, an open approach was chosen due to anticipated adhesions from prior surgery. A combined repair was performed.

An open transabdominal approach was undertaken through a lower midline infraumbilical incision. The bladder was exposed extraperitoneally and then bivalved vertically in the midline from dome to bladder neck, providing excellent access to the posterior bladder wall and trigone.

A supra-trigonal fistula located on the left posterolateral wall was identified. The fistulous tract was carefully dissected free from surrounding scar tissue. The vaginal defect was closed first in a single layer using interrupted absorbable sutures. Subsequently, the bladder wall was closed in two watertight layers, ensuring mucosal inversion. An omentum/peritoneal flap was interposed between the bladder and vaginal closures to reinforce the repair.

The right ureter, involved in a concomitant ureterovaginal fistula, was dissected and mobilised proximally to healthy tissue. It was reimplanted into the bladder via ureteroneocystostomy using the modified Lich-Gregoir technique, over a double-J stent.

Low-pressure continuous drainage was achieved with both a Foley catheter and suprapubic catheter. The abdominal incision was closed in layers.

Postoperatively, the patient was hemodynamically stable with no febrile episodes or signs of infection; recovery was uneventful. She received intravenous fluids, appropriate analgesia, and prophylactic antibiotics as per protocol. On postoperative day (POD) 3, a pelvic closed-suction Jackson-Pratt drain, adjacent to the repair site, had minimal serous output and was removed.

The Foley catheter was retained for 14 days, while the suprapubic catheter was maintained for 21 days to ensure adequate bladder drainage. During the postoperative period, the patient was encouraged to mobilize early and was provided with chest physiotherapy along with incentive spirometry to promote optimal pulmonary function and recovery. There was no evidence of pericatheter leakage. Abdominal wound dressing and perineal hygiene were maintained as per hospital infection control protocol.

Follow-up ultrasound performed prior to discharge showed no significant hydronephrosis, and renal function tests remained within normal limits. The patient was discharged in stable condition on POD 5 with advice on adequate hydration, catheter care, and scheduled follow-up for stent removal and reassessment of urinary continence and renal drainage. Ureteric stent removal was scheduled for six weeks postoperatively.

Ureteric stent removed at six weeks. At six-month follow-up, the patient remained continent with normal renal function and no hydronephrosis. Long-term follow-up is necessary to monitor for any potential recurrences.

## Discussion

VVFs and UVFs pose a significant challenge to urologists, particularly in resource-limited settings [4]. This is a case report of a 36-year-old woman suffering from VVF concomitant with UVF and with a clinical presentation of an urge to void and pass 100-150 ml of urine during normal voiding. This clinical presentation is commonly found in UVF cases [10]. Continuous urinary leak is the most frequent symptom in 76.4% of UVF cases [4]. In our patient, urinary incontinence was noted by POD 10, which aligns with the literature's incidence of 23.85 ± 19.17 days post-surgery [4].

Key diagnostic tools that can assist in identifying an early UVF include contrast-enhanced CT urography, which helps to visualise the course of the ureter and identify extravasation of contrast [5]. Intravenous urography (IVU) is helpful for evaluating ureteral patency and contrast extravasation into abnormal cavities [5]. Cystoscopy with retrograde pyelography helps visualise the bladder and ureters and identify fistulous tracts [8]. Indocyanine green (ICG) fluorescence ureteral assessment is a newer technique to detect subtle ureteral injuries [11]. The methylene blue test is used intraoperatively by instilling dye into the bladder and confirming any leakage [12].

Intraoperative vigilance is crucial. Surgeons should ensure bilateral ureteral patency by performing retrograde dye studies or placing ureteral stents to confirm the integrity of the ureters. Any leak should warrant a thorough investigation to diagnose an underlying UVF.

The hazards of missing an associated UVF

Failure to identify a concomitant UVF in a patient with VVF has serious repercussions. If the fistula remains undiagnosed and untreated, continuous urinary leakage will persist, vexing both the patient and the treating physician [4]. Prolonged urinary leakage from unrecognised UVF can lead to complications such as:

Need for Second Surgery

If UVF is unrecognised after primary VVF repair, the patient will need an additional procedure that can be significantly more complex due to post-surgical fibrosis, altered anatomical planes, and increased technical difficulty in identifying and accessing the affected ureter.

Hydroureteronephrosis and Renal Dysfunction

Persistent urine leakage leads to urine accumulation in the affected ureter, causing progressive ureteral dilatation and fibrosis. If left unrecognised, it can potentially result in permanent renal damage.

Recurrent UTI

Chronic leakage predisposes patients to recurrent UTIs, further worsening patients’ morbidity.

Worsening Quality of Life

Despite undergoing a technically successful VVF repair, the patient will continue to suffer, with urinary leakage causing persistent social and emotional distress [6,11].

Need for an aggressive diagnostic approach

Failing to diagnose and manage concomitant UVF can lead to complications such as deteriorated renal function [4], urinary tract infection (UTI), abnormal genital function, and urosepsis.

The literature evidences a delayed approach to UVF repair mainly related to abdominal or vaginal hysterectomies [9,11]. The delay in fistulae repair may be attributed to nonspecific symptoms and delayed presentation of urinary leakage [4], high-volume hospitals [12], and other reasons such as personal inconvenience, emotional distress [13], and social stigma [4].

In about 44% of UVF cases, intervention was delayed, and this was attributed to a delay in the recording of symptoms after the operation and the social stigma attached to urine leakage [4].

The combined approach of right ureteric reimplantation with VVF repair was practical in resolving the patient’s symptoms. Ureteric reimplantation is a time-proven and effective treatment for UVFs if conventional approaches like stenting are implausible [2]. Simple ureteric reimplantation was observed to be the most common treatment modality in about 28.6% of UVF patients [4], and a similar report is found in individual case reports [10]. However, the procedure has a drawback of reflux after reimplantation [14]. In line with the present study, complicated VVF concurrent with UVF was managed by primary ureteral reimplantation [15].

The European Association of Urology guidelines suggest stent placement as the treatment modality for UVF [16]. A pooled cohort showed a 95% success rate for the stent if done within two weeks of fistula detection. In addition to the short intervention time, the success of stenting depends on the presence of a nonoccluded ureter and injury site within 2 cm from the ureteric orifice [6,17].

In case of concurrent VVF or consistent urine leakage despite stent placement, surgical treatment with ureteric implantation is suggested. Ureteric reimplantation by the laparoscopic method showed low morbidity, shortened hospital stay, fewer analgesic treatments, and a higher success rate in UVF patients [18]. A shorter operative time with less blood loss is indicated in case of an early ureteral reimplantation [19], whereas, in a pooled study, irrespective of the timing of intervention, ureteric implantation was reported to have a 100% success rate in the treatment of UVF [4].

However, we opted for the open approach in our patient due to the anticipation of dense adhesions from the prior procedure.

Take-home message

Given the severe consequences of missed diagnosis of UVF, an aggressive and systematic approach to diagnose this “critical dyad” is imperative in all patients presenting with VVF. A comprehensive diagnostic workup should always be performed to assess the integrity of the ureters and bladder.

## Conclusions

Concomitant VVF and UVF present significant diagnostic and therapeutic challenges. Although VVF is often the primary concern, the potential presence of an occult UVF must not be overlooked. Given the risk of serious complications, all patients with VVF should undergo thorough evaluation to exclude UVF. Early identification and combined surgical management of both fistulas in a single session can reduce morbidity, avoid secondary procedures, and preserve renal function. This case highlights how systematic preoperative assessment - using multimodal imaging and intraoperative ureteral evaluation - enabled definitive simultaneous repair, preventing reoperation and ensuring optimal long-term outcomes.
